# Association of a Multifaceted Intervention With Ordering of Unnecessary Laboratory Tests Among Caregivers in Internal Medicine Departments

**DOI:** 10.1001/jamanetworkopen.2019.7577

**Published:** 2019-07-24

**Authors:** Renuka S. Bindraban, Marlou van Beneden, Mark H. H. Kramer, Wouter W. van Solinge, Peter M. van de Ven, Christiana A. Naaktgeboren, Muhammad Al-Dulaimy, Lena C. van der Wekken, Yvonne C. Bandt, Frank Stam, Suzanne I. M. Neppelenbroek, Anita Griffioen-Keijzer, Daan A. R. Castelijn, Brigitte A. Wevers, Anneroos W. Boerman, Merel van Wijnen, Maarten J. ten Berg, Prabath W. B. Nanayakkara

**Affiliations:** 1 Section of Acute Medicine, Department of Internal Medicine, Amsterdam Public Health Research Institute, Amsterdam UMC, Vrije Universiteit Amsterdam, Amsterdam, the Netherlands; 2 Department of Clinical Chemistry and Hematology, University Medical Center Utrecht, Utrecht, the Netherlands; 3 Department of Epidemiology and Biostatistics, Amsterdam UMC, Vrije Universiteit Amsterdam, Amsterdam, the Netherlands; 4 Julius Center for Health Sciences and Primary Care, University Medical Center Utrecht, Utrecht, the Netherlands; 5 Department of Internal Medicine, Zaans Medical Center, Zaandam, the Netherlands; 6 Department of Clinical Pharmacy, Zaans Medical Center, Zaandam, the Netherlands; 7 Department of Internal Medicine, North-West Hospital Group, Alkmaar, the Netherlands; 8 Department of Internal Medicine, Spaarne Gasthuis, Haarlem, the Netherlands; 9 Department of Internal Medicine, Spaarne Gasthuis, Hoofddorp, the Netherlands; 10 Atalmedial Medical Diagnostics Centers, Hoofddorp, the Netherlands; 11 Department of Internal Medicine, Meander Medical Center, Amersfoort, the Netherlands; 12 Department of Clinical Chemistry, Meander Medical Center, Amersfoort, the Netherlands

## Abstract

**Importance:**

Inappropriate use of laboratory testing is a challenging problem. Estimated overuse rates of approximately 20% have been reported. Effective, sustainable solutions to stimulate optimal use are needed.

**Objective:**

To determine the association of a multifaceted intervention with laboratory test volume.

**Design, Setting, and Participants:**

A before-after quality improvement study was performed between August 1, 2016, and April 30, 2018, in the internal medicine departments of 4 teaching hospitals in the Netherlands. Data on laboratory order volumes from 19 comparable hospitals were used as controls. The participants were clinicians ordering laboratory tests.

**Interventions:**

The intervention included creating awareness through education and feedback, intensified supervision of residents, and changes in order entry systems. Interventions were performed by local project teams and guided by a central project team during a 6-month period. Sustainability was investigated during an 8-month follow-up period.

**Main Outcomes and Measures:**

The primary outcome was the change in slope for laboratory test volume. Secondary outcomes were change in slope for laboratory expenditure, order volumes and expenditure for other diagnostic procedures, and clinical outcomes. Data were collected on duration of hospital stay, rate of repeated outpatient visits, 30-day readmission rate, and rate of unexpected prolonged duration of hospital stay for patients admitted for pneumonia.

**Results:**

The numbers of internists and residents ordering tests in hospitals 1 to 4 were 16 and 30, 18 and 20, 13 and 17, and 21 and 60, respectively. Statistically significant changes in slope for laboratory test volume per patient contact were found at hospital 1 (change in slope, −1.55; 95% CI, −1.98 to −1.11; *P* < .001), hospital 3 (change in slope, −0.74; 95% CI, −1.42 to −0.07; *P* = .03), and hospital 4 (change in slope, −2.18; 95% CI, −3.27 to −1.08; *P* < .001). At hospital 2, the change in slope was not statistically significant (−0.34; 95% CI, −2.27 to 1.58; *P* = .73). Laboratory test volume per patient contact decreased by 11.4%, whereas the volume increased by 2.4% in 19 comparable hospitals. Statistically significant changes in slopes for laboratory costs and volumes and costs for other diagnostic procedures were also observed. Clinical outcomes were not associated with negative changes. Important facilitators were education, continuous attention for overuse, feedback, and residents’ involvement. Important barriers were difficulties in data retrieval, difficulty in incorporation of principles in daily practice, and high resident turnover.

**Conclusions and relevance:**

A set of interventions aimed at changing caregivers’ mindset was associated with a reduction in the laboratory test volume in all departments, whereas the volume increased in comparable hospitals in the Netherlands. This study provides a framework for nationwide implementation of interventions to reduce unnecessary laboratory testing.

## Key Points

**Question:**

What is the association of a multifaceted intervention aimed at changing the mindset of caregivers with the amount of unnecessary laboratory testing?

**Findings:**

In this before-after quality improvement study conducted in the internal medicine departments of 4 large teaching hospitals in the Netherlands, the volume of laboratory tests ordered per patient contact was reduced in all 4 departments by 11.4% overall. In contrast, volume increased by 2.4% in 19 comparable hospitals in the Netherlands.

**Meaning:**

The approach used in this study can be used as a framework for future projects aiming to reduce unnecessary laboratory diagnostic tests in routine clinical practice.

## Teaser

This before-after quality improvement study evaluates whether a multifactorial intervention was associated with a reduction in the volume of laboratory tests ordered by clinicians in the internal medicine departments of teaching hospitals in the Netherlands.

## Introduction

In recent years, the concept of low-value care has gained attention, and international campaigns have been launched to discourage the unnecessary use of tests and procedures. In the United Kingdom, Do Not Do recommendations^1^ were formulated, and in the United States, the Choosing Wisely campaign was introduced.^2^ Thereafter, many other countries, including the Netherlands, followed suit with programs aimed at deimplementing unnecessary care.^3^

Inappropriate use of laboratory tests is a well-recognized phenomenon, and estimated overuse rates of approximately 20% have been reported.^4^ Overuse is also reflected in high interphysician variability of test orders. In a recent study^5^ among internal medicine residents, some residents ordered 7 to 8 times more tests than their peers. Many interventions have proven effective in reducing unnecessary laboratory testing.^6,7,8^ In addition to financial consequences, overuse is less patient friendly and may increase the number of false-positive results, which leads to more, potentially harmful tests.^9^

In 2012, our study group published the findings of a multifaceted intervention^10^ begun in 2008 aimed at reducing unnecessary diagnostic testing through increasing awareness at the Internal Medicine Department of the Vrije Universiteit Medical Center in Amsterdam, the Netherlands. Although we mainly focused on laboratory testing, the use of other diagnostic procedures also decreased. A 13% gross reduction in diagnostic expenditure was observed compared with the previous year and was sustained over subsequent years.^11^

In the Reduction of Unnecessary Diagnostics Through Attitude Change of the Caregivers (RODEO) project, we implemented this same multifaceted intervention in the internal medicine departments of 4 large teaching hospitals in the Netherlands. The goal of this project was to reduce total laboratory testing by 5%. Our primary focus was on laboratory testing, although associations with other diagnostic procedures were also assessed. Furthermore, we assessed the facilitators and barriers to deimplementation of unnecessary testing.

## Methods

This project is a part of the To Do or Not to Do? Reducing Low-Value Care program,^12^ a national program initiated by the Dutch Federation of University Medical Centers. The study protocol providing a detailed description of the methods has been published previously.^13^ Therefore, we provide only a brief overview of the methods here.

This report follows the Standards for Quality Improvement Reporting Excellence (SQUIRE) reporting guideline for quality improvement studies. The medical ethics review committee of Vrije Universiteit Medical Center assessed the project protocol, determined that official approval by the committee was not required, and waived the need for informed consent because data were collected anonymously. Local ethics committees and boards of directors of all participating hospitals approved the study.^13^

### Study Design and Setting

We conducted a before-after quality improvement study at the departments of internal medicine of 4 teaching hospitals in the Netherlands. The study was performed by a coordinating project team together with a local project team at each hospital (eAppendix 1 in the Supplement). The inclusion criteria were previously described.^13^

### Characteristics of Participating Departments

Table 1 shows the characteristics of participating departments at initiation of the project. The departments differ mainly in annual patient load and in number of physicians working at the departments. In the remainder of this article, the participating hospitals are referred to as hospitals 1 to 4. Numbers were selected randomly.

**Table 1.** Characteristics of Participating Departments at Initiation of the Project

Characteristic	Zaans Medical Center	North-West Hospital Group, Location Alkmaar	Spaarne Gasthuis, Locations Haarlem and Hoofddorp	Meander Medical Center
Annual emergency department visits for internal medicine, No.	3000	3800	6000	4400
Annual outpatient department visits for internal medicine, No.	25 000	36 900	54 200	37 600
Annual inpatient admissions for internal medicine, No.	1800	3000	4248	2900
Internists, No.	13	18	21	16
Residents, No.	17	20	60	30
Involvement of clinical chemist	Participation in clinical meetings once a month, available on call	No participation in clinical meetings, available on call	Participation in several clinical meetings, available on call	Participation in clinical meetings including daily morning report, available on call
Laboratory ordering system	Electronic	Electronic at emergency department and inpatient clinic, paper forms at outpatient department	Electronic	Electronic at emergency department and inpatient clinic, electronic and paper forms at outpatient clinic
Comments	In the preceding years the Zaans Medical Center already actively focused on reducing unnecessary care through several projects and initiatives	The Medical Center Alkmaar and the Gemini Hospital in Den Helder merged in 2015	The Kennemer Gasthuis in Haarlem and Spaarne Hospital merged in 2015	Send-out test requests are discussed with clinicians before approval

### Outcomes, Data Sources, and Measurements

Our primary outcome was change in slope for laboratory test volume per patient contact. Secondary outcomes were change in slope for laboratory test costs; order volumes and costs for radiology, microbiology, and nuclear medicine tests per patient contact; and clinical outcomes. Orders placed for the internal medicine specialty by inpatient and outpatient departments and the emergency department were included.

To investigate whether our intervention influenced patient care, we assessed the mean duration of hospital stay, rate of repeated outpatient visits, 30-day readmission rate, and rate of unexpected prolonged duration of hospital stay for patients admitted for pneumonia. The last 2 outcomes are quality indicators assessed yearly by the Healthcare Inspectorate in the Netherlands.^14^

In addition, we collected data on laboratory order volumes from 19 comparable hospitals in the Netherlands. Most of these were large peripheral hospitals, reflecting the national mix of hospital type. The criterion for inclusion was availability of data for the duration of the project. The benchmark data used in this study are based on standardized and validated production data of these of 19 hospitals. These data were acquired through Performation, a Dutch data-driven consultancy firm.^15^

### Assessment of Facilitators and Barriers

Facilitators and barriers were identified through questionnaires with the project teams^13^ at initiation, during joint conferences, and through a questionnaire filled out by all physicians at participating departments at the end of the project (eAppendix 2 in the Supplement).

### Deimplementation Strategy

**Figure 1.**
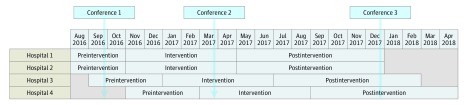
Project Timeline at 4 Hospitals in the Netherlands, August 1, 2016, Through April 30, 2018

A timeline of the project is shown in Figure 1. After consent by the board of directors of each hospital, cooperation agreements were signed. Thereafter, project teams consisting of 1 or more internists, residents, and clinical chemists and a business intelligence or control specialist were formed.

#### Preintervention Period

Ordering patterns from the preceding years were analyzed with the aim of recognizing patterns in test use. A joint conference was organized with all members of the project teams to exchange ideas and experiences and to discuss target laboratory items for reduction. In addition, anticipated facilitators and barriers were discussed. Each department was encouraged to develop interventions specifically for their department, in addition to the generic intervention that is described in this article.

#### Intervention Period

The local project teams performed the interventions and had monthly meetings with the coordinating project team, during which the progress of the study and ordering patterns were discussed. A second joint conference was organized with all project teams to discuss interim results, facilitators and barriers, and ideas and experiences.

#### Postintervention Period

A third joint conference was organized, during which the main goal was to discuss sustainability. The local project teams continued the actions introduced earlier, performed new actions, and had 2 to 3 monthly progress meetings with the coordinating project team.

#### Description of Interventions

The intervention consisted of several items. Education and feedback were used to create awareness and were provided through, for example, presentations and newsletters. In addition, supervision of residents by experienced physicians regarding test ordering was intensified. This entailed explicitly focusing on indications for tests and asking critical questions during morning reports and other clinical meetings. In addition, modifications were made to order entry systems, for example by enacting time limits on repeated orders.

The actions performed in each clinic are displayed in eFigure 1 in the Supplement. Details on specific actions are published in the study protocol.^13^ An overview of the modifications made to the order entry systems is provided in eTable 1 in the Supplement.

The project teams placed specific focus on a set of tests that are known to be frequently overused: blood urea nitrogen, creatinine, amylase, aspartate aminotransferase, alanine aminotransferase, C-reactive protein, and erythrocyte sedimentation rate. These were used to create a change in physician mindset, which we believed would lead to greater awareness when requesting diagnostic procedures in general. A more-detailed description of the deimplementation strategy is published in the protocol.^13^

### Statistical Analysis

#### Volumes and Costs of Diagnostic Procedures

For our primary outcome, we collected the weekly number of tests performed during the intervention and postintervention period and the 2 preceding years. Secondary outcomes were reported by the hospitals per month. We adjusted volumes and costs for patient load using the number of patient contacts, defined as the sum of the number of visits, day admissions, and patient days for the internal medicine department. We decided against using standardized patient units to adjust for patient load, which we previously described in the study protocol,^13^ because we observed that a large proportion of diagnostic procedures were ordered during repeated outpatient visits that were not included in the calculation of standardized patient units.

#### Clinical Outcomes

The mean duration of hospital stay and rate of repeated outpatient visits were assessed monthly. The 30-day readmission rate and the rate of unexpected prolonged duration of hospital stay for patients admitted for pneumonia were assessed yearly for 2015, 2016, and 2017.

#### Analysis

All statistical analyses were performed using the forecast package in R statistical software version 3.4.3 (R Project for Statistical Computing). Interrupted time series analyses were performed using the weekly or monthly data to assess the associations of the intervention with volumes and costs. We used an autoregressive integrated moving average model to analyze whether a (more profound) change in the number or costs of tests per patient contact was observed after starting the intervention. We adjusted for seasonal variation using autocorrelations with a yearly period. The trend in mean was modeled using a regression model with separate parameters for slope before and after implementation of the intervention and allowing for a direct change in the period after the start of the intervention. The autocorrelation and moving average parameters were selected using the automated auto.arima function in R, minimizing the corrected Akaike information criterion statistic. Outcomes are expressed as the difference in slope between the 2 years before the intervention started and the 14 months after the intervention started (6-month intervention period plus 8-month postintervention period). We used a *z *test to determine whether regression coefficients in the autoregressive integrated moving average model differed from 0. All *P* values refer to 2-sided tests. *P* < .05 was considered statistically significant. The percentage change in laboratory test volume per patient contact was calculated using the mean number of laboratory tests per patient contact during the final 6 months of the project and the mean number during the same 6 months of the previous year.

## Results

The numbers of internists and residents ordering tests in hospitals 1 to 4 were 16 and 30, 18 and 20, 13 and 17, and 21 and 60, respectively.

### Laboratory Test Volume

**Figure 2.**
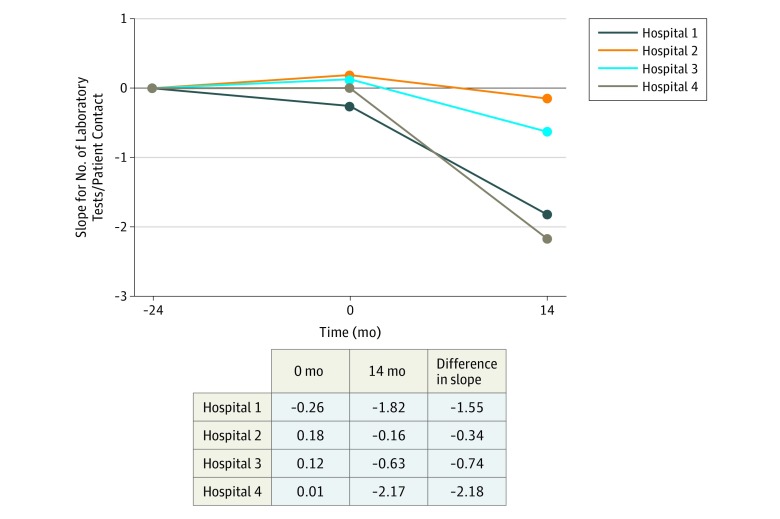
Slopes and Differences for Laboratory Test Volume Before and After the Start of the Intervention^a^ ^a^The intervention included creating awareness through education and feedback, intensified supervision of residents, and changes in ordering entry systems.

Our main goal was to assess the association of our multifaceted intervention with laboratory test volume. Slopes and changes in slopes for laboratory test volume per patient contact per year are presented in Figure 2 and Table 2. Changes in slope were statistically significant at hospital 1 (−1.55; 95% CI, −1.98 to −1.11; *P* < .001), hospital 3 (−0.74; 95% CI, −1.42 to −0.07; *P* = .03), and hospital 4 (−2.18; 95% CI, −3.27 to −1.08; *P* < .001). At hospital 2, the change in slope was not statistically significant (−0.34; 95% CI, −2.27 to 1.58; *P* = .73). For hospitals 1 to 4, reductions in numbers of tests per patient contact in the last 6 months of the intervention were 11.5%, 5.9%, 8.3%, and 14.7%, respectively, compared with the same period in the year before, yielding an overall decrease of 11.4%. In eFigures 2, 3, 4, and 5 in the Supplement, we present the changes in laboratory test volumes relative to the same months in the preceding year during the 14 months, along with the interventions performed.

**Table 2.** Changes in Volumes and Costs for Diagnostics Following the Intervention^a^

Variable	Hospital 1		Hospital 2		Hospital 3		Hospital 4	
	Change (95% CI)	*P* Value	Change (95% CI)	*P* Value	Change (95% CI)	*P* Value	Change (95% CI)	*P* Value
Volume								
Laboratory	−1.55 (−1.98 to −1.11)	<.001	−0.34 (−2.27 to 1.58)	.73	−0.74 (−1.42 to −0.07)	.03	−2.18 (−3.27 to −1.08)	<.001
Radiology	−0.03 (−0.05 to −0.01)	.005	0.01 (−0.02 to 0.03)	.57	0 (−0.02 to 0.03)	.80		
Microbiology	−0.02 (−0.07 to 0.11)	.62	0.15 (0.02 to 0.28)	.02	−0.16 (−0.28 to −0.03)	.02		
Nuclear medicine	0 (−0.02 to 0.02)	.99	−0.04 (−0.05 to −0.02)	<.001	−0.02 (−0.03 to −0.01)	<.001		
Costs								
Laboratory	−4.13 (−9.26 to 1.01)	.12	−0.90 (−4.17 to 2.37)	.59	−1.88 (−4.4 to 0.67)	.14	−8.60 (−12.94 to −4.25)	<.001
Radiology	4.57 (1.61 to 7.53)	.002	1.42 (−0.72 to 3.56)	.20	1.49 (−0.19 to 3.17)	.08		
Microbiology	−0.08 (−1.41 to 1.26)	.91	0.66 (−0.66 to 1.98)	.33	−1.91 (−4.53 to 0.71)	.14		
Nuclear medicine	−4.96 (−9.11 to −0.81)	.02	−14.26 (−21.19 to −7.33)	<.001	−0.35 (−3.27 to 2.57)	.81		

^a^ Expressed as change in slope for number of tests per patient contact per year (for volume), or costs for tests in euros per patient contact per year (for costs). For hospital 4, data on radiology, microbiology, and nuclear medicine were not available.

#### Control Group

In the departments of internal medicine of 19 comparable hospitals, we saw a significant increase of 0.32 laboratory tests per patient contact per year (95% CI, 0.21-0.42; *P* < .001) between January 1, 2015, until our intervention started in the first 2 hospitals on November 1, 2016, and a comparable increase (0.33; 95% CI, 0.20-0.47; *P* < .001) between November 1, 2016, and April 30, 2018, which was when the project was concluded. The number of laboratory tests per patient contact increased by 2.4% in the last 6 months of the project compared with the same period in the year before.

#### Specific Tests

The departments targeted all tests described in the Methods section, except for hospital 2, which did not focus on blood urea nitrogen and creatinine. Significant changes in slope for number of requests were observed for blood urea nitrogen and creatinine (hospitals 1 and 4), amylase (hospitals 1, 2, and 3), aspartate aminotransferase (hospitals 3 and 4), and alanine aminotransferase (hospital 4). Full data are shown in eTable 2 in the Supplement. The associations of the multifaceted intervention with costs of laboratory tests, and with volumes and costs of radiology, microbiology, and nuclear medicine tests are presented in Table 2.

### Clinical Outcomes

Clinical outcomes are shown in Table 3. For mean duration of hospital stay, no significant changes in slope were observed. For rate of repeated outpatient visits, a significant decreasing change in slope was found at hospital 1. The 30-day readmission rates remained unchanged. The rate of unexpected prolonged duration of hospital stay for patients admitted with pneumonia increased in all departments, which was consistent with the national trend and presumably was attributable to the severe influenza epidemic in 2017.

**Table 3.** Clinical Outcomes

Outcome	Hospital 1		Hospital 2		Hospital 3		Hospital 4	
	Value	*P* Value	Value	*P* Value	Value	*P* Value	Value	*P* Value
LOS, mean^a^	−0.60 (−1.57 to 0.36)	.22	0.23 (−0.49 to 0.96)	.53	−0.13 (−1.20 to 0.94)	.81	0.44 (−0.19 to 1.06)	.17
Repeat frequency^a^	−0.40 (−0.72 to 0.09)	.01	−0.38 (−0.90 to 0.14)	.15	−0.09 (−0.40 to 0.22)	.58	0.07 (−1.03 to 1.16)	.90
30-d readmissions^b^								
2015	12%		97		104		110	
2016	13%		98		107		117	
2017	13%		100		90		111	
Unexpected prolonged LOS^c^								
2015	87		11.6%		16.7%		14.6%	
2016	93		12.9%		15.2%		13.1%	
2017	97		15.8%		18.9%		15.2%	

Abbreviation: LOS, length of stay.

^a^ Expressed as change in slope (95% CI).

^b^ Thirty-day readmissions are expressed as either the percentage of total admissions that were readmissions within 30 days, or as a ratio (total number of 30-day readmissions/expected number of 30-day readmissions according to case mix).

^c^ Unexpected prolonged LOS is expressed as either a percentage of total admissions for pneumonia with a LOS greater than or equal to 150% than the mean LOS during the previous year, or as a ratio (number of admissions for pneumonia with LOS greater than or equal to 150% than the mean LOS during the previous year/number of admissions for pneumonia with LOS greater than or equal to 150% than the mean LOS during the previous year, expected according to case mix).

### Facilitators and Barriers to Deimplementation

The factors identified through the initial questionnaire and first and second conference, categorized into different levels as proposed by Grol and Wensing,^16^ were used as input to adjust our strategy (eTable 3 in the Supplement). During the final conference and through the final questionnaire, we evaluated which factors were of greatest influence (eTables 4, 5, and 6 in the Supplement).

Important facilitators were education, continuous attention for diagnostic testing, and feedback. Involvement of clinical chemists and establishing clear working agreements were also considered important. During the project, the teams were expanded with physicians representing internal medicine subspecialties. This facilitated our efforts to obtain widespread support, which was a crucial element in the RODEO project. Having enthusiastic internists and residents function as role models was considered a strong facilitating factor. Members of the coordinating project team viewed the involvement of residents as the main factor contributing to the success of the project.

Although the teams aimed to establish clear working agreements, incorporation in daily practice was impeded by the high rate of turnover of residents, which required regular repetition of RODEO principles. The most important barrier was obtaining reliable data on order volumes and costs, which made it difficult to monitor progress in some clinics. In addition, it took several months before reduction efforts translated into consistent changes in ordering patterns (eFigures 2, 3, 4, and 5 in the Supplement). It was challenging to maintain the efforts needed for the project, especially when only 1 resident was included in the project team and that resident changed rotation. Moreover, we noticed that lessening attention was associated with an almost immediate change in ordering patterns (eFigures 2, 3, 4, and 5 in the Supplement). Although modifying order systems was an important facilitator, their rigidness was seen as a barrier in 1 hospital, because clinicians were not immediately informed when a test was not performed and the time limits for repeated requests were considered too strict.

## Discussion

In this project, we aimed to reduce inappropriate laboratory testing by implementing interventions aimed at changing the mindset of health care professionals. In 3 of 4 departments, a significant decrease in slope was found for laboratory test volume, whereas an increase in slope was observed in 19 comparable hospitals in the Netherlands. The laboratory test volume per patient contact decreased by 11.4% overall. Although every department performed interventions from all categories, nuances were different. In hospital 1, the role of the clinical chemist in the project team was substantial, whereas hospital 3 mainly focused on order system changes by instituting time limits for repeat orders, and hospital 4 placed emphasis on educating physicians.

For other diagnostic procedures, significant changes in slopes were also found. At hospital 1, statistically significant changes in slope were found for volume and/or costs of laboratory, radiology, and nuclear medicine tests. At hospital 2, although a 5.9% reduction in volume of laboratory tests per patient contact was achieved, the change in slope was not statistically significant. For nuclear medicine, we did find statistically significant decreasing changes in slope for volume and costs, which we think are a result of the departments’ increased focus on indications for positron emission tomography scans during the intervention period. At hospital 3, the decreases in slope for order volumes of microbiology and nuclear medicine tests were statistically significant, together with laboratory test volume. For hospital 4, data on diagnostic procedures other than laboratory tests were not available. The observed changes in ordering patterns for diagnostic procedures on which little focus was placed suggest that the intervention led to a change in caregivers’ mindset for ordering diagnostic procedures in general. Clinical outcomes were not associated with negative changes following the reduction in diagnostic testing.

Overall, important facilitators to deimplementation were education, feedback, continuous attention for diagnostic testing, and involvement of residents. In contrast to hospitals 3 and 4, only 1 resident was included in the project team at hospital 2. In addition, this resident changed rotation during the project. Also, the outpatient department at hospital 2 was not as emphatically targeted, although we observed that in all participating hospitals, a large number of orders were placed at the outpatient department. We believe that this finding, in part, reflects the importance of obtaining widespread support for the success of the project. This knowledge was used as input for performing the project at hospitals 3 and 4, where several residents were included in the local project team from initiation, and much attention was paid to orders placed at the outpatient department. The most important barriers were difficulties in data collection, difficulties in incorporation of working agreements in daily practice, and a high rate of resident turnover.

The total cost for performing the project was approximately €250 000. The largest part of this amount was spent on the personnel who coordinated the project (€200 000). At the end of the project, the intervention was integrated into daily routine without hiring extra staff. As for potential cost reduction through reducing laboratory testing, an estimated reduction of approximately €1.2 million could be made. This yields an 11.4% reduction of the total €10.9 million spent on laboratory testing in 2017 by all 4 departments. Because of the fixed costs for personnel and laboratory equipment, the actual cost reduction will be lower. Cost reduction through the reduction of other diagnostic procedures as well as downstream costs were not included in this calculation.

### Comparison With Previous Literature

In line with previous literature,^6,7,8^ a multifaceted intervention was associated with a reduction in laboratory testing. Contrary to most other studies, we used a multicenter approach, introducing our intervention in 4 large hospitals. Data were collected during the 6-month intervention period and, unlike most other studies, we also assessed sustainability during an additional 8-month period. Furthermore, we collected clinical data to investigate patient-related outcomes. This study is one of the first to investigate all these aspects, to our knowledge, and we further demonstrate that with the help of local teams, these interventions can be implemented successfully in daily practice. Previous literature^6,7,8^ does not show that any particular intervention is the most effective, and combined interventions are advocated. However, in the questionnaires conducted at the end of the project, the physicians in our project reported perceiving the educational sessions to be the most effective intervention, followed by modifications in order systems.

### Strengths and Limitations

The intervention used in this study was originated and first applied in an academic medical center in Amsterdam.^10,11^ In the current study, we have shown that it is feasible to implement this intervention in 4 large peripheral teaching hospitals. The positive and sustained changes observed in different settings suggest that our approach may also be effective in other hospitals and for other services. By assessing facilitators and barriers, we point out specific issues to take into account in future studies. Another strength entails assessment of sustainability. Although we investigated short-term sustainability, long-term sustainability can be expected through our approach, as we have previously shown.^11^ Measures to ensure sustainability include, for example, repeated education, modifications in ordering systems, posters stating important principles regarding test ordering displayed in work spaces, mouse pads with reminders regarding test ordering, and inclusion of these principles in the introductory program for new employees. Another strength of this project is the study of clinical outcomes. Another strength involves the intervention itself. By providing departments the liberty to place focus on different elements of the standard intervention, we ensured that the actions suited the departmental structure.

This project has several limitations. First, because multiple interventions were performed at once, it was not possible to assess the effectiveness of individual interventions. Second, data collection was difficult and incomplete for 1 hospital. Also, it was not possible to assess the numbers of blood samples obtained at all clinics, which would be a desirable end point in future studies in the context of delivering patient-friendly care. The lack of patient involvement in this project is another limitation.

## Conclusions

A set of interventions aimed at changing caregivers’ mindset was associated with a reduction in the laboratory test volume in all departments, whereas the volume increased in comparable hospitals in the Netherlands. In 3 of 4 departments, the change in slope was significant. Laboratory costs and other diagnostic procedures were also reduced following the intervention, and clinical outcomes were unchanged. Furthermore, we identified facilitators and barriers to deimplementation. The approach used in this study can be extended to other types of services and clinics. This study provides a framework for nationwide implementation of these interventions and might be complemented with involving patients and emphasizing patient-friendly care in future efforts.

## Notes

Supplement. **eAppendix 1.** Project Teams **eAppendix 2. **RODEO Project Evaluation **eFigure 1. **Interventions Divided by Category **eTable 1. **Redundancy Checks **eFigure 2. **Hospital 1: Interventions and Laboratory Test Volumes Relative to Preceding Year **eFigure 3. **Hospital 2: Interventions and Laboratory Test Volumes Relative to Preceding Year **eFigure 4. **Hospital 3: Interventions and Laboratory Test Volumes Relative to Preceding Year **eFigure 5. **Hospital 4: Interventions and Laboratory Test Volumes Relative to Preceding Year **eTable 2. **Effect of Interventions on Ordering of Specific Tests **eTable 3. **Facilitators and Barriers: Questionnaire Willingness to Change, First and Second Conference **eTable 4. **Facilitators and Barriers: Third Conference **eTable 5. **Facilitators: Questionnaire RODEO Project Evaluation **eTable 6.** Barriers: Questionnaire RODEO Project Evaluation
