# Supplementary material for: Association of a Multifaceted Intervention With Ordering of Unnecessary Laboratory Tests Among Caregivers in Internal Medicine Departments
Source: JAMA Netw Open. 2019 Jul 24;2(7):e197577. doi: 10.1001/jamanetworkopen.2019.7577 (PMC6659142; doi:10.1001/jamanetworkopen.2019.7577)
Supplement: Supplement. — eAppendix 1. Project Teams eAppendix 2. RODEO Project Evaluation eFigure 1. Interventions Divided by Category eTable 1. Redundancy Checks eFigure 2. Hospital 1: Interventions and Laboratory Test Volumes Relative to Preceding Year eFigure 3. Hospital 2: Interventions and Laboratory Test Volumes Relative to Preceding Year eFigure 4. Hospital 3: Interventions and Laboratory Test Volumes Relative to Preceding Year eFigure 5. Hospital 4: Interventions and Laboratory Test Volumes Relative to Preceding Year eTable 2. Effect of Interventions on Ordering of Specific Tests eTable 3. Facilitators and Barriers: Questionnaire Willingness to Change, First and Second Conference eTable 4. Facilitators and Barriers: Third Conference eTable 5. Facilitators: Questionnaire RODEO Project Evaluation eTable 6. Barriers: Questionnaire RODEO Project Evaluation [file jamanetwopen-2-e197577-s001.pdf]

Supplementary Online Content

Bindraban RS, van Beneden M, Kramer MHH, et al. Association of a multifaceted intervention with ordering of unnecessary laboratory tests among caregivers in internal medicine departments. *JAMA Netw Open*. 2019;2(7):e197577. doi:10.1001/jamanetworkopen.2019.7577

eAppendix 1. Project Teams

eAppendix 2. RODEO Project Evaluation

eFigure 1. Interventions Divided by Category

eTable 1. Redundancy Checks

eFigure 2. Hospital 1: Interventions and Laboratory Test Volumes Relative to Preceding Year

eFigure 3. Hospital 2: Interventions and Laboratory Test Volumes Relative to Preceding Year

eFigure 4. Hospital 3: Interventions and Laboratory Test Volumes Relative to Preceding Year

eFigure 5. Hospital 4: Interventions and Laboratory Test Volumes Relative to Preceding Year

eTable 2. Effect of Interventions on Ordering of Specific Tests

eTable 3. Facilitators and Barriers: Questionnaire Willingness to Change, First and Second Conference

eTable 4. Facilitators and Barriers: Third Conference

eTable 5. Facilitators: Questionnaire RODEO Project Evaluation

eTable 6. Barriers: Questionnaire RODEO Project Evaluation

This supplementary material has been provided by the authors to give readers additional information about their work.

eAppendix 1: Project Teams

Principal investigator
Dr. P.W.B. Nanayakkara, MD, PhD, FRCP. Section Acute Medicine, Department of Internal Medicine, Amsterdam Public Health Research Institute, Amsterdam UMC, Vrije Universiteit Amsterdam , The Netherlands

Coordinating investigators
Drs. R.S. Bindraban, MD. Department of Clinical Chemistry and Hematology, University Medical Center Utrecht, Utrecht, The Netherlands. Section Acute Medicine, Department of Internal Medicine, Amsterdam Public Health Research Institute, Amsterdam UMC, Vrije Universiteit Amsterdam , The Netherlands

Drs. M.L.H. van Beneden, MSc. Section Acute Medicine, Department of Internal Medicine, Amsterdam Public Health Research Institute, Amsterdam UMC, Vrije Universiteit Amsterdam , The Netherlands

Investigators
Dr. M.J. ten Berg, PharmD, PhD. Department of Clinical Chemistry and Hematology, University Medical Center Utrecht, Utrecht, The Netherlands

Prof. dr. W.W. van Solinge, PhD. Department of Clinical Chemistry and Hematology, University Medical Center Utrecht, Utrecht, The Netherlands

Prof. dr. M.H.H. Kramer, MD, PhD, FRCP. Section Acute Medicine, Department of Internal Medicine, Amsterdam Public Health Research Institute, Amsterdam UMC, Vrije Universiteit Amsterdam , The Netherlands

Advisors coordinating project team
Dr. C.A. Naaktgeboren, PhD. Department of Clinical Chemistry and Hematology, University Medical Center Utrecht, Utrecht, The Netherlands. Julius Center for Health Sciences and Primary Care, University Medical Center Utrecht, The Netherlands.

Dr. P.M. van de Ven, PhD. Department of Epidemiology and Biostatistics, Amsterdam UMC, Vrije Universiteit Amsterdam, The Netherlands

Local project teams
Zaans Medical Center, Zaandam, The Netherlands
M. Al-Dulaimy, Department of Internal Medicine
L.C. van der Wekken, Department of Internal Medicine
J.W. Plaisier, Department of Internal Medicine
Y.C. Bandt, Department of Clinical Pharmacy
N. Osmanovic, Laboratory of Clinical Chemistry
J. Dinkelaar, Laboratory of Clinical Chemistry

North-West Hospital Group, location Alkmaar, The Netherlands
F. Stam, Department of Internal Medicine
B. van Dam, Department of Internal Medicine
D. ten Oever, Department of Internal Medicine
S.I.M. Neppelenbroek, Department of Internal Medicine
V.A. de Weger, Department of Internal Medicine
E. ten Boekel, Department of Clinical Chemistry, Hematology and Immunology
J. de Gans - de Wit, Department of Business Control

Spaarne Gasthuis, locations Hoofddorp and Haarlem, The Netherlands
A. Griffioen – Keijzer, Department of Internal Medicine
R. Soetekouw, Department of Internal Medicine
K .Farhat, Department of Internal Medicine
D.A.R. Castelijn, Department of Internal Medicine
J.S. ten Kulve, Department of Internal Medicine
N.N. Radhakishun, Department of Internal Medicine
C.I.M. de Roij van Zuijdewijn, Department of Internal Medicine
N. Slager, Department of Planning and Control

M.M. Buijs, Atalmedial Medical Diagnostics Centers, Hoofddorp, the Netherlands
B.A. Wevers, Atalmedial Medical Diagnostics Centers, Hoofddorp, the Netherlands

Meander Medical Center, Amersfoort, The Netherlands
R. Fijnheer, Department of Internal Medicine
A.W. Boerman, Department of Internal Medicine
R. Goedegebuure, Department of Internal Medicine
J.A.J. Traa, Department of Finance and Control
M. van Wijnen, Department of Clinical Chemistry

© 2019 Bindraban RS et al. *JAMA Network Open.*

eAppendix 2. RODEO Project Evaluation

Male / Female
Resident / Internist
Number of years of experience within this function
For how long have you been employed at this department?
Have you contributed to the project? If yes, how did you contribute?

During the past months, we have carried out the RODEO project within your department, in which we aimed to reduce the amount of unnecessary diagnostics without affecting quality of care. Through this questionnaire we would like to assess your thoughts on the RODEO project. We ask for your opinion on and experiences with unnecessary diagnostics, and specifically, we ask questions regarding aspects that were addressed in this project. Please return the filled in questionnaire to us before February 28th 2018.

Fully agree, agree, neutral, disagree, fully disagree, not applicable

1. The importance of reducing unnecessary diagnostics was clear.
2. The aim of the project was clear.
3. Enough attention was paid to the importance for patients of reducing unnecessary diagnostics.
4. The environment at the department was such that I felt free to ask questions regarding the usefulness of test requests.
5. The amount of questions colleagues have asked me regarding the usefulness of test requests was sufficient.
6. Internists have asked me, as resident, a sufficient amount of questions regarding the usefulness of test requests.
7. As internist, I have asked residents a sufficient amount of questions regarding the usefulness of test requests.
8. I have been sufficiently informed about the progress of the project.
9. (Changes in) ordering patterns at department level have been made sufficiently transparent.
10. Reducing unnecessary testing has been sufficiently supported by scientific evidence.
11. There was sufficient space to bring in ideas for the project.
12. I have gained new knowledge on diagnostics.
13. Novel working agreements have been sufficiently embedded into daily practice.
14. Reducing unnecessary diagnostics leads to higher quality care.
15. Reducing unnecessary diagnostics leads to more patient friendly care.
16. I fear to miss clinically relevant information by performing less diagnostic tests.
17. During the past month, I have received negative feedback for performing less diagnostic tests.

Never, <1x/month, 1x/month, 1x/3 weeks, 1x/2 weeks, 1x/week, >1x/week

18. Before the project, how often did you see examples of unnecessary use of diagnostics?
19. After the project, how often do you see examples of unnecessary use of diagnostics?
20. How often was unnecessary use of diagnostics addressed during morning or afternoon reports?
21. How often was unnecessary use of diagnostics addressed during grand rounds?
22. How often was unnecessary use of diagnostics addressed during other clinical discussions?
23. How often has time been reserved explicitly for discussion of unnecessary use of diagnostics?

Open-ended questions

24. Which interventions (addressing unnecessary use during clinical meetings, education, changes in order entry systems, feedback on ordering patterns, involvement of clinical chemist, etc.) did you find most effective?
25. Which factors were facilitators of the project?
26. Which factors were barriers to the project?
27. How could the agreements made in this project be sustained?
28. Do you have any further tips or comments?

© 2019 Bindraban RS et al. *JAMA Network Open.*

eFigure 1. Interventions Divided by Category

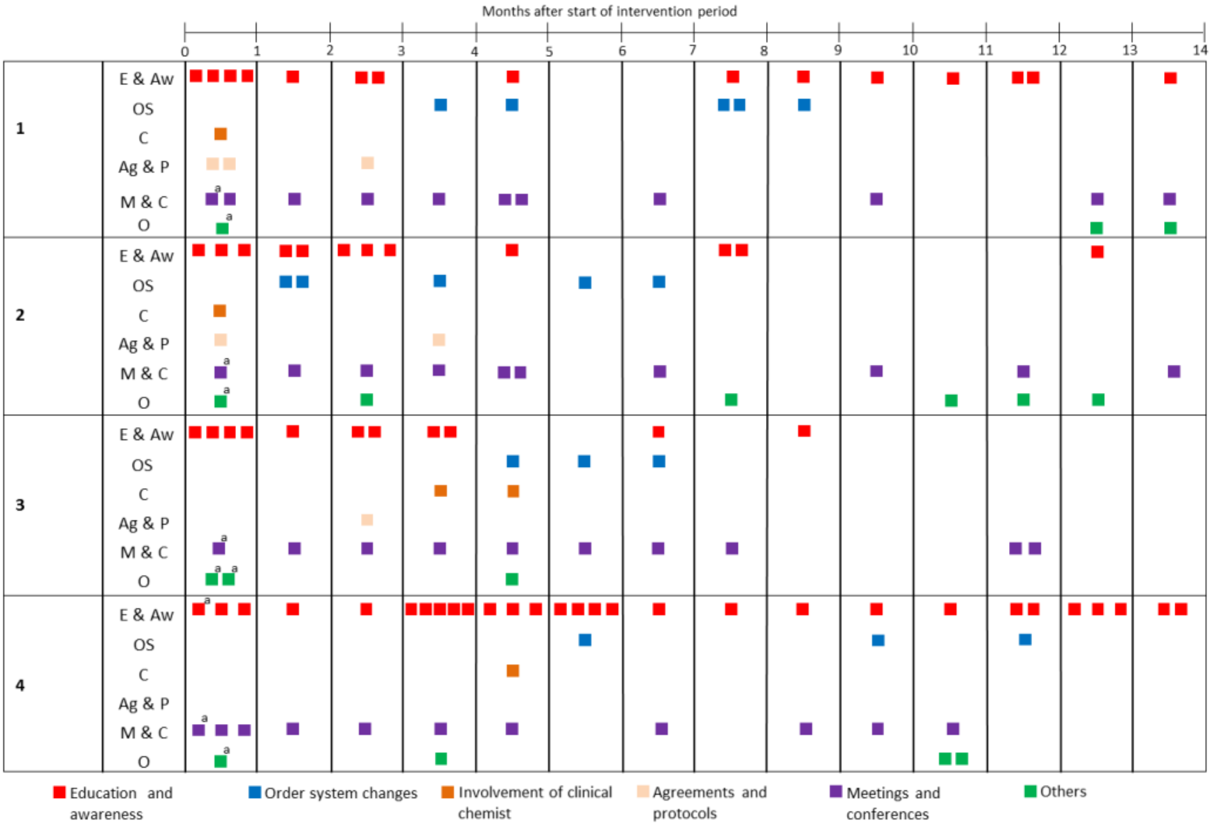

[a]: Action took place before intervention period

© 2019 Bindraban RS et al. *JAMA Network Open.*

A Multicenter Before-After Study on Reducing Unnecessary Diagnostics by Changing the Attitude of Caregivers: Protocol for the RODEO Project. Bindraban RS, van Beneden ML, Kramer MH, van Solinge WW, Neppelenbroek SI, van Wijnen M, Griffioen-Keijzer A, Al-Dulaimy M, Ten Berg MJ, Nanayakkara PW. JMIR Res Protoc. 2018 Aug 21;7(8):e10473

© 2019 Bindraban RS et al. *JAMA Network Open.*

eTable 1. Redundancy Checks

	Hospital 1		Hospital 2		Hospital 3		Hospital 4	
**Laboratory test**	**Present at initiation**	**Present at conclusion**	**Present at initiation**	**Present at conclusion**	**Present at initiation**	**Present at conclusion**	**Present at initiation**	**Present at conclusion**
1,25-hydroxy vitamin D	21 days	21 days						
25-OH vitamin D	21 days	28 days						
Alkaline phosphatase						Inp. 1 day; Outp. 2 days[a]		
ALT						Inp. 1 day; Outp. 2 days[a]		
AST						Inp. 1 day; Outp. 2 days[a]		5 days
Amylase						Inp. 1 day; Outp. 7 days[a]		Pop-up upon request[b]
Anti-phospholipid antibodies	56 days	56 days						
Alpha-1-antitrypsin						Once[a]		
Alpha-1-antitrypsine in feces	14 days	14 days						
ANA			30 days	30 days				
Anti-cardiolipin			70 days	70 days				
Anti-CCP			150 days	150 days		365 days		
Apolipoprotein A1	14 days	14 days						
Apolipoprotein B	14 days	14 days						
Bilirubin						Inp. 1 day; Outp. 2 days[a]		
BNP	5 days	5 days	7 days	7 days				
CDT	14 days	14 days						
Chromosome test	Once	Once						
Creatinin						Inp. 1 day; Outp. 3 days[a]		
CRP						Inp. 1 day; Outp. 5 days[a]		2 days

© 2019 Bindraban RS et al. *JAMA Network Open.*

ESR		4 days	1 day	< 18 y, 1 day; >18 y, 7 days		7 days[a]		

	Hospital 1		Hospital 2		Hospital 3		Hospital 4	
**Laboratory test**	**Present at initiation**	**Present at conclusion**	**Present at initiation**	**Present at conclusion**	**Present at initiation**	**Present at conclusion**	**Present at initiation**	**Present at conclusion**
Ferritin				7 days		14 days[a]		
Folic acid		28 days		20 days		30 days[a]		
Free protein S	30 days	30 days						
FT4				> 18 y, 5 days		30 days		
GGT						Inp. 1 day; Outp. 2 days[a]		
HbA1c	21 days	21 days	40 days	40 days		30 days		
HDL cholesterol				14 days		30 days[a]		
IgA								
IgG						1 year, unless abnormal		
IgM						1 year, unless abnormal		
Iron						14 days[a]		
Iron-binding capacity						14 days[a]		
Iron saturation						14 days[a]		
Irregular antibodies	3 days	3 days						
Lactate dehydrogenase						Inp. 7 days; Outp. 14 days		
LDL cholesterol				14 days		30 days[a]		
Leukocyte differential count		1 day	1 day	1 day			3 days	3 days
Lipoprotein (a)	14 days	14 days						
Lupus anticoagulans			70 days	70 days				
NT-proBNP						30 days		
p-Elastase in feces	30 days	30 days						
Protein C Activity	30 days	30 days						
Protein C Antigen	30 days	Abolished						
Protein S Antigen	30 days	Abolished						
Rheumatoid factor			30 days	30 days				

© 2019 Bindraban RS et al. *JAMA Network Open.*

	Hospital 1		Hospital 2		Hospital 3		Hospital 4	
**Laboratory test**	**Present at initiation**	**Present at conclusion**	**Present at initiation**	**Present at conclusion**	**Present at initiation**	**Present at conclusion**	**Present at initiation**	**Present at conclusion**
Serum protein	30 days	7 days	5 days	20 days				
Total cholesterol						30 days[a]		
Triglycerides						Inp. 5 days; Outp. 30 days[a]		
TSH				> 18 y, 5 days		30 days		
T3				> 18 y, 5 days		30 days		
Tumor markers (PSA, CEA, CA-125, CA15-3, AFP, b-HCG)						14 days[a]		
Urea								5 days
Viscosity	7 days	Abolished						
Vitamin A	7 days	7 days						
Vitamin B2	7 days	7 days						
Vitamin B12		28 days				30 days[a]		
Vitamin E	7 days	7 days						
Vitamins			7 days	14 days				
Zinc protoporphyrin	30 days	Abolished						

[a] The time limit for repetitive requesting of this test was adjusted during the project due to resistance from physicians working at the department. [b] Pop-up instated upon each request: For pancreatitis, amylase testing is not deemed appropriate at this hospital. Lipase is sufficient. Abbreviations: ANA, antinuclear antibodies; CDT, carbohydrate deficient transferrin; ESR, erythrocyte sedimentation rate; FT4, free thyroxin; GGT, gamma-glutamyl transferase; TSH, thyroid stimulating hormone; PSA, prostate specific antigen; CEA, carcino-embryonic antigen; CA-125, cancer antigen-125, CA15-3, cancer antigen 15-3; AFP, alpha fetoprotein; b-HCG, beta-human chorionic gonadotropin; Inp, inpatient department; Outp, outpatient department.

© 2019 Bindraban RS et al. *JAMA Network Open.*

eFigure 2. Hospital 1: Interventions and Laboratory Test Volumes Relative to Preceding Year

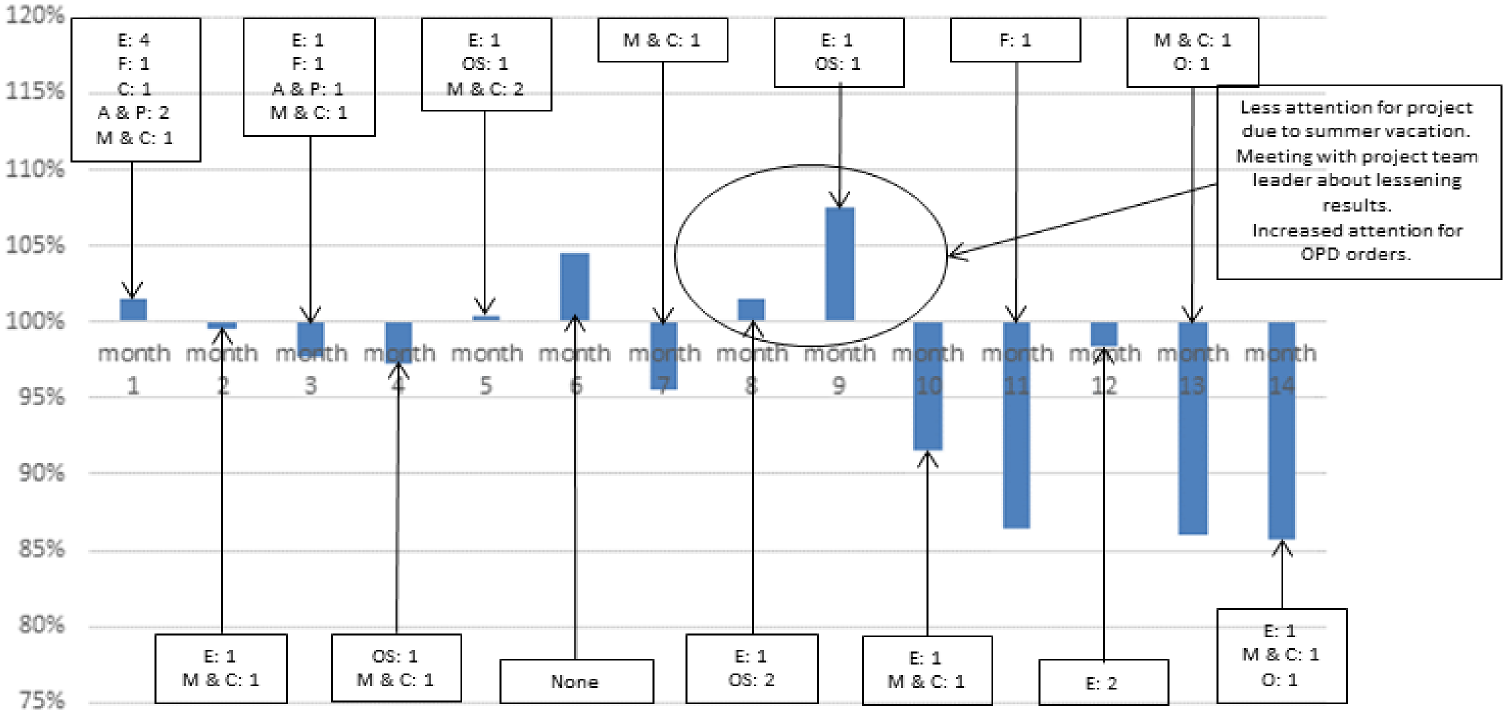

Figure legend: E: Education; OS: Order system changes; C: Involvement of clinical chemist; Ag & P: Agreements and protocols; M & C: Meetings & conferences; O: Others

eFigure 3. Hospital 2: Interventions and Laboratory Test Volumes Relative to Preceding Year

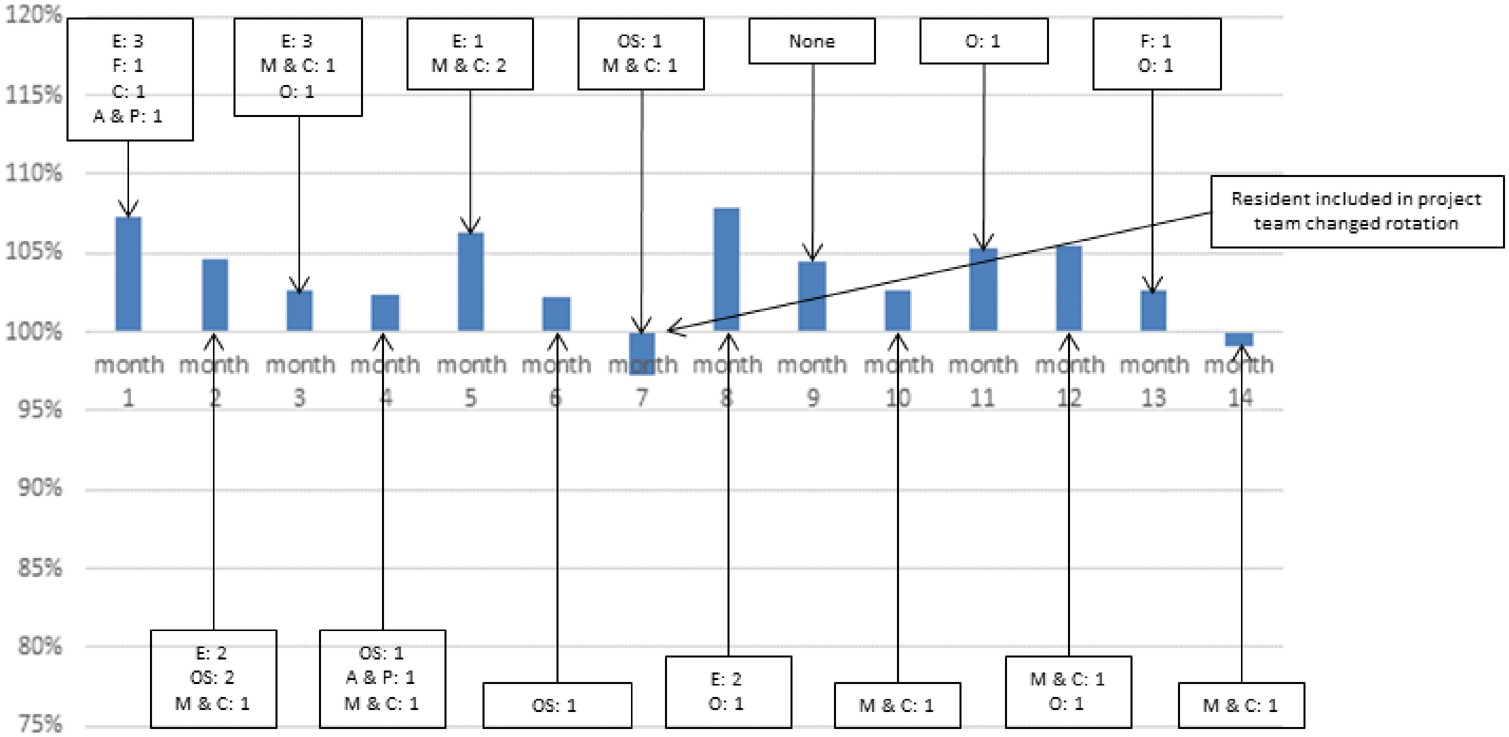

Figure legend: E: Education; OS: Order system changes; C: Involvement of clinical chemist; Ag & P: Agreements and protocols; M & C: Meetings & conferences; O: Others

© 2019 Bindraban RS et al. *JAMA Network Open.*

eFigure 4. Hospital 3: Interventions and Laboratory Test Volumes Relative to Preceding Year

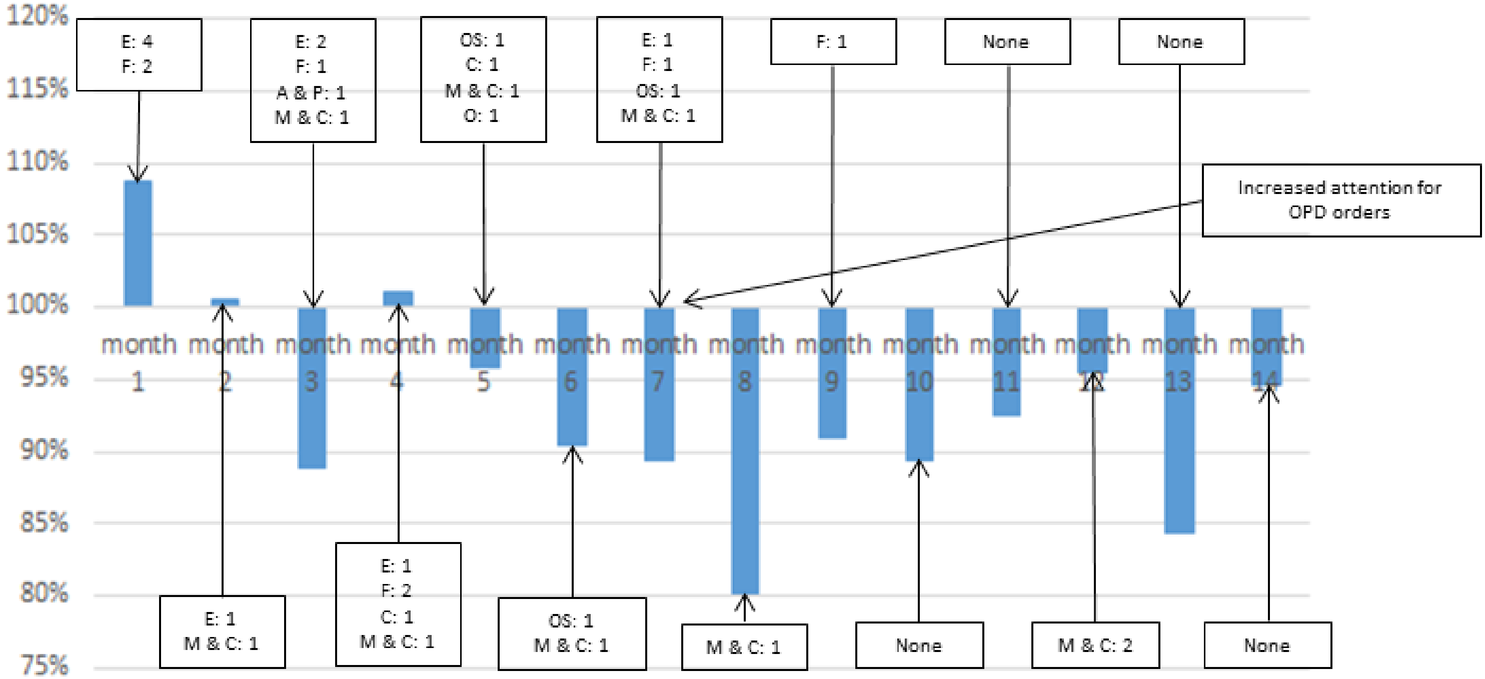

Figure legend: E: Education; OS: Order system changes; C: Involvement of clinical chemist; Ag & P: Agreements and protocols; M & C: Meetings & conferences; O: Others

eFigure 5. Hospital 4: Interventions and Laboratory Test Volumes Relative to Preceding Year

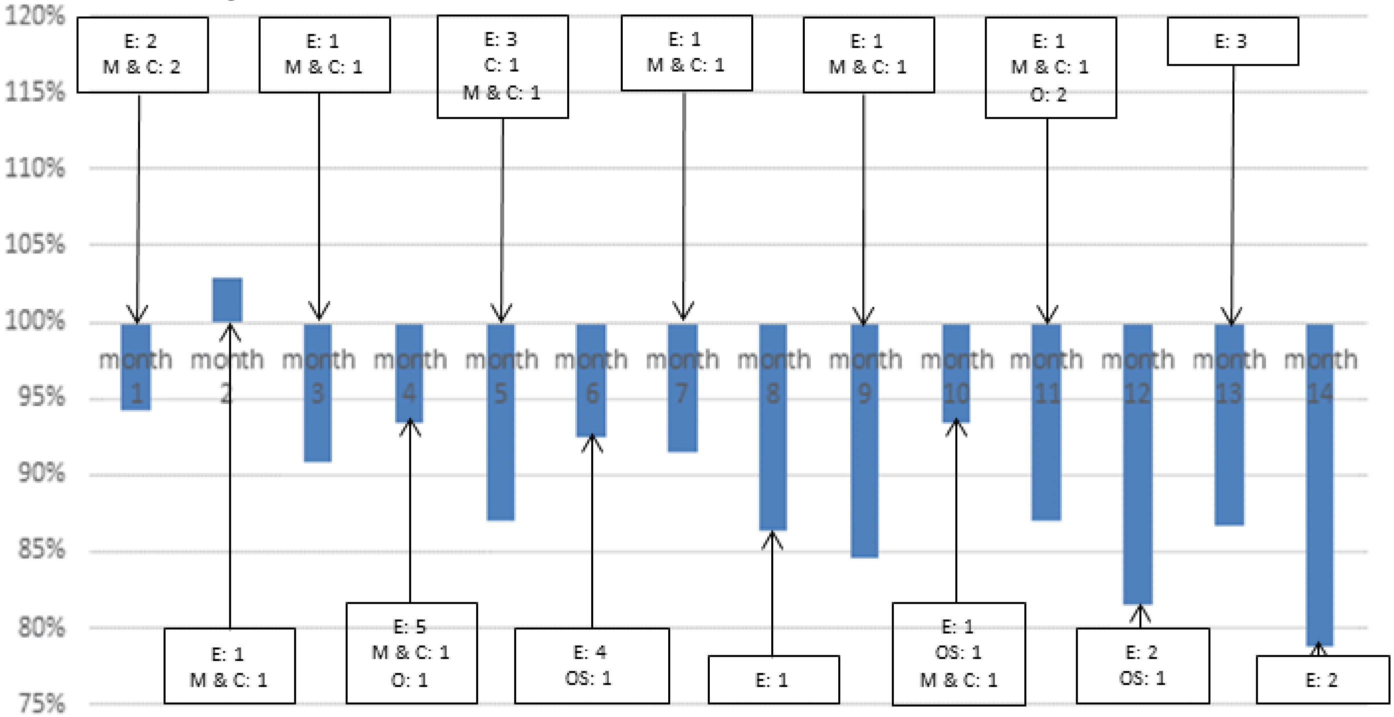

Figure legend: E: Education; OS: Order system changes; C: Involvement of clinical chemist; Ag & P: Agreements and protocols; M & C: Meetings & conferences; O: Others

© 2019 Bindraban RS et al. *JAMA Network Open.*

eTable 2. Effect of Interventions on Ordering of Specific Tests

	Hospital 1			Hospital 2			Hospital 3			Hospital 4		
**Test**	**Change**	**95% CI**	**p**	**Change**	**95% CI**	**p**	**Change**	**95% CI**	**p**	**Change**	**95% CI**	**p**
BUN	-0.08	-0.11, -0.05	< 0.001	0.00	-0.01, 0.02	0.69	0.00	-0.03, 0.04	0.84	-0.07	-0.12, -0.02	0.01
Creatinin	-0.07	-0.12, -0.02	0.01	+0.01	-0.02, 0.04	0.41	-0.02	-0.07, 0.03	0.40	-0.13	-0.20, -0.06	< 0.001
Amylase	-0.01	-0.02, -0.01	< 0.001	-0.05	-0.07, -0.04	< 0.001	+0.05	0.04, 0.06	< 0.001	+0.01	-0.03, 0.05	0.62
AST	-0.02	-0.03, 0.002	0.07	+0.01	-0.003, 0.02	0.14	-0.13	-0.17, -0.10	< 0.001	-0.07	-0.10, -0.05	< 0.001
ALT	-0.01	-0.02, 0.01	0.58	+0.01	-0.001, 0.02	0.07	-0.02	-0.05, 0.01	0.27	-0.07	-0.09, -0.04	< 0.001
CRP	0.00	-0.02, 0.03	0.72				+0.03	-0.002, 0.06	0.07	-0.03	-0.06, 0.01	0.09
ESR							-0.01	-0.02, 0.002	0.1	0.00	-0.01, 0.02	0.46

Change in slope for volumes of specific laboratory tests. Changes are expressed as increase of decrease in number of tests per patient contact per year. Abbreviations: BUN, blood urea nitrogen; AST, aspartate aminotransferase; ALT, alanine aminotransferase; CRP, C-reactive protein; ESR, erythrocyte sedimentation rate. For hospitals 1 and 2, not all data was available

© 2019 Bindraban RS et al. *JAMA Network Open.*

eTable 3. Facilitators and Barriers: Questionnaire Willingness to Change, First and Second Conference

	Facilitators	Barriers
**Individual factors: caregivers**	- Educational efforts, supported by scientific evidence<br>- Continuous attention for overuse, repetition<br>- Enthusiasm caregivers and project teams<br>- Importance for health care and resident training<br>- Focusing on quality and safety	- Fear of missing clinically relevant information, insecurity<br>- Fear of prolongation of length of stay<br>- Ambiguity about what overuse reduction efforts yield<br>- Difficult to change working habits or routine behavior<br>- Costs are negligible and remain vague
**Individual factors: patients**	- Involvement of patients in efforts to reduce overuse	
**Social factors**	- Making use of morning reports, grand rounds, etc. to discuss ordering behavior<br>- Role models, local champions<br>- Providing insight into ordering patterns and costs<br>- Feedback on progress and changes in ordering patterns<br>- Establishing clear agreements on ordering diagnostics<br>- Involvement of subspecialties to broaden support<br>- Involvement of clinical chemists and controllers	- Lack of role models<br>- Physicians don't feel personally responsible for making changes<br>- Difficulty in establishing clear agreements on ordering diagnostics<br>- Lack of consensus among specialists<br>- Unwillingness of specialists
**Organizational factors**	- Changes in ordering systems<br>- In-depth evaluation of ordering patterns<br>- Simple dataset for follow-up<br>- Feasibility within department<br>- Sustainability of results<br>- Incorporation of RODEO and its principles into introduction programs for new employees	- Lack of time and availability of physicians, clinical chemists and controllers to dedicate to the project<br>- High rate of turnover of residents<br>- Convenience of standard ordering panels<br>- Difficulties in obtaining correct utilization data

© 2019 Bindraban RS et al. *JAMA Network Open.*

	- Support by coordinating project team<br>- Hospital-wide introduction of RODEO	- Long lead time to implement changes in ordering systems<br>- Changes in ordering systems can often only be made for the entire hospitals, thus requiring consensus between specialties<br>- Fear Department of Clinical Chemistry for an increase in the number of afterwards requested tests
**Environmental factors**	- Reducing overuse is currently a hot topic	- Focusing on euros instead of on quality and safety<br>- Willingness of diagnostics departments might be affected by negative effects on their income

eTable 4. Facilitators and Barriers: Third Conference

	**Facilitators**	**Barriers**
**Individual factors: caregivers**	- Educational efforts, supported by scientific evidence<br>- Continuous attention for overuse, repetition<br>- Enthusiasm caregivers and project teams	
**Individual factors: patients**	- Involvement of patients in efforts to reduce overuse	
**Social factors**	- Making use of morning reports, grand rounds, etc. to discuss ordering behavior<br>- Role models, local champions<br>- Involvement of subspecialties to broaden support	- Lack of communication
**Organizational factors**	- Changes in ordering systems<br>- Simple dataset for follow-up	- Performing too many actions at once<br>- Placing too much focus on details

© 2019 Bindraban RS et al. *JAMA Network Open.*

	- Incorporation of RODEO and its principles into introduction programs for new employees<br>- Incentivize controller	
**Environmental factors**		- Focusing on cost reduction instead of on quality and safety

© 2019 Bindraban RS et al. *JAMA Network Open.*

eTable 5. Facilitators: Questionnaire RODEO Project Evaluation (n = 76)

**Facilitators**		Hospital 1	Hospital 2	Hospital 3	Hospital 4	Total
Individual factors: caregivers	- Educational efforts, supported by scientific evidence	8	3	1	4	16
	- Continuous attention for overuse	6	3	2	0	11
	- Enthusiasm of caregivers and project teams	1	2	1	2	6
Social factors	- Feedback on progress and changes in ordering patterns	5	1	3	0	9
	- Involvement of clinical chemists	5	0	0	0	5
	- Involvement of residents	0	3	1	0	4
	- Results of the project	0	1	0	1	2
	- Involvement of project team	0	0	0	1	1
	- Involvement of internists	0	0	1	0	1
	- Initiating the project	0	0	1	0	1
Organizational factors	- Order system systems	4	0	0	0	4
	- Clarity about the aims of the project	1	0	0	0	1
Environmental factors	- Not focusing on cost reduction	0	0	1	0	1
	- Reducing overuse is currently a hot topic	0	1	0	0	1

eTable 6. Barriers: Questionnaire RODEO Project Evaluation (n = 76)

**Barriers**		Hospital 1	Hospital 2	Hospital 3	Hospital 4	Total
Individual factors: caregivers	- Lack of attention for overuse reduction	2	0	0	0	2
	- Difficult interpretability of results	1	0	0	0	1
Social factors	- Principles and agreements not fully incorporated in day-to-day practice	3	0	0	1	4
	- Lack of feedback	1	1	0	0	2
	- Role of supervisor	2	0	0	0	2
	- Lack of involvement of clinical chemists	1	0	0	0	1
	- Lack of presence of specialists at educational sessions concerning their specialty	1	0	0	0	1
	- Lack of visibility of project	0	0	1	0	1
	- Reproachful tone of clinical chemistry department	0	0	1	0	1
Organizational factors	- High rate of turnover of residents	1	1	0	2	4
	- Rigidness of order system changes	0	0	0	4	4
	- Lack of time or other priorities	0	1	1	1	3

© 2019 Bindraban RS et al. *JAMA Network Open.*

	- Difficulties in instatement of order system changes, not enough order system changes<br>- Difficulties in obtaining correct utilization data<br>- Logistics	0<br><br>0<br>0	1<br><br>2<br>0	0<br><br>0<br>0	2<br><br>0<br>1	3<br><br>2<br>1
Environmental factors	- Focusing on cost reduction	0	0	1	0	1

© 2019 Bindraban RS et al. *JAMA Network Open.*
